# The effectiveness of hippotherapy simulation exercises for muscle strength, disease activity and quality of life in sedentary adults with ankylosing spondylitis

**DOI:** 10.1080/07853890.2023.2249822

**Published:** 2023-08-29

**Authors:** Ender Salbaş, Ali Yavuz Karahan

**Affiliations:** a Physical Medicine and Rehabilitation, Bandirma Training and Research Hospital, Balikesir, Turkey; b Physical Medicine and Rehabilitation, Usak University, Usak, Turkey

**Keywords:** Exercise therapy, devices, hippotherapy simulation, muscularity, quality of life, Registration ID: NCT04651725

## Abstract

**Introduction/Objective:**

Newly created systems called hippotherapy simulators (HS) mimic the primitive movements of a live horse. As they are new systems, research examining their usefulness has been well received. The aim of this study is to research the effects of HS on disease activity, quality of life and muscle strength in patients with ankylosing spondylitis (AS).

**Methods:**

In a prospective, assessor-blinded, block-randomized trial, 48 AS patients were randomly assigned in a 1:1 ratio to receive either HS or conventional home (CH) exercise therapy. All Participants received 48 sessions, that is 4 sessions a week for 12 consecutive weeks. The primary outcome measures included the quadriceps muscle strength, Bath Ankylosing Spondylitis Disease Activity Index (BASDAI), Bath Ankylosing Spondylitis Functional Index (BASFI), Bath Ankylosing Spondylitis Metrology Index (BASMI) and Ankylosing Spondylitis Quality of Life Scale (ASQoL).

**Results:**

Both groups demonstrated significant improvement in BASDAI, BASFI, BASMI, ASQoL and muscle strength scores compared to the baseline (*p* < 0.05). BASDAI, BASFI and BASMI scores decreased significantly in the HS group compared to the CH group at week 12 (*p*=.005, *p*=.003, *p*=.045, respectively), but there was no significant difference between the groups in terms of ASQoL and muscle strength scores at week 12 (*p*=.245, *p*=.212, respectively).

**Conclusions:**

The results of this clinical trial of HS exercises for AS patients indicate a positive effect on disease activity, quality of life and muscle strength. Therefore, horse-riding simulator exercises can be used as an alternative method for the management of individuals with AS.

## HIGHLIGHTS

- Hippotherapy simulator exercises indicate a positive effect on disease activity, functionality and muscle strength in people with ankylosing spondylitis
- For people with ankylosing spondylitis, hippotherapy simulator exercises are a safe workout for the spine that uses the core muscles.
- For people with ankylosing spondylitis, the hippotherapy simulator technique can be recommended as a long-lasting and affordable fitness program in the near future.
- To determine the efficacy of hippotherapy simulation exercise on other systemic chronic inflammatory disease future research are needed.

## 1. Introduction

People with Ankylosing Spondylitis (AS) experience joint stiffness and pain that mainly affects the spine, hips and shoulders [1,2]. Since the cause is an inflammatory condition, morning stiffness and pain can be prominent symptoms [1,2]. Inflammation of the ligament or joint capsule and enthesitis in the areas where tendons attach to bone are the main pathomechanisms of this syndrome, causing new bone formation and ankylosis [1–3]. Therefore, combinations of different anti-inflammatory drugs are an option in the management of ankylosing spondylitis [1–3]. Furthermore, similar to other chronic musculoskeletal diseases such as osteoarthritis, clinical guidelines recommend therapeutic exercises and physical activities to relieve pain and stiffness [4]. Exercise therapy can effectively improve musculoskeletal function and health-related quality of life in people with AS. Despite the fact that AS can affect muscle strength, balance and cardio-respiratory function, the majority of published data focuses on mobility exercise, with little emphasis on other exercise domains such as strengthening, balance or cardio-respiratory exercise [4,5]. According to the Cochrane Review published by Regnaux et al. there is moderate-to-low-quality evidence to suggest that therapeutic exercises improve physical function, reduce pain and may slightly reduce participants’ global assessments of disease activity compared to the no-intervention group [6]. Furthermore, in the existing literature, specific exercise programs for AS vary in terms of types, such as Pilates, yoga, tai chi, water exercises and exergames [6–14]. Similarly, there is a variety of information on the dosage (duration, frequency and intensity) of therapeutic programs [4,6,7]. In conclusion, there is consensus that ‘the most effective therapeutic exercise approach remains unclear’ [4,6]. Being a traditional goal for patients with AS, however, a balanced exercise program has focused on improving physical function and maintaining posture. According to Sarac et al. loss of postural stability, low physical activity, and increased fatigue were associated with a decrease in core muscle endurance. The authors of this study suggested that prescribed exercises could improve core strength in the AS population [15]. Therefore, the main components that should be included in the daily exercise routine of individuals with AS should be mobility exercises for the axial skeleton and peripheral joints, anti-gravity muscle strengthening, stretching methods and cardiorespiratory fitness [4,6–14].

Horseback riding is recommended as a form of exercise as it has been shown to offer therapeutic advantages [16,17]. Hippotherapy has been shown in many studies to help stabilize muscles and improve core strength, as well as increase circulatory function, promote balance and enhance overall health [16,17]. The horse’s four-beat gait involves multidirectional movements and this pattern is used in physiotherapy for postural control, increasing range of motion, balance, strengthening and stretching [16,17]. Because of these characteristics, hippotherapy is used to treat a wide range of conditions, including cerebral palsy, Down syndrome, muscular dystrophy, cerebrovascular disease, multiple sclerosis (MS), spinal cord disorders, autistic behavioral disorders, psychiatric disorders, rheumatic diseases and low back pain [18–21]. However, this type of exercise requires additional safety precautions. Even if the horses used for this purpose are very safe and well trained, it is possible for them to spook or become nervous. An experienced therapist is essential to carry out the activity effectively and safely, and constant care must be taken to ensure that the patient does not fall off the horse [18–21]. There are also a few other limitations, such as allergies to horses or the environment and the cost of the treatment. Newly created systems called hippotherapy simulators (HS) mimic the primitive movements of a live horse. As they are a new system, research examining their usefulness has been well received, especially in children with cerebral palsy, stroke, multiple sclerosis and geriatric patients [22–25]. Such repetitive movements improve postural rhythm and coordination, allow reciprocal movement and promote postural control by triggering balancing reactions. To our knowledge, there are no current studies examining the effects of exercises with HS in AS patients.

The aim of this study is to research the effects of HS on disease activity, quality of life and muscle strength in adults with AS. Therefore, the null hypothesis of this study states that exercises with HS will have no effect on disease activity, quality of life and muscle strength in adults with AS.

## Patients and the method

### Design and participants

This prospective study was conducted as a randomized trial with parallel groups in a single-center. The setting of this quantitative study was the outpatient clinic of the physical medicine and rehabilitation department of a university hospital (University of Usak (Turkey)) between September 2020 and February 2022.

After the trial was approved by the Ethical Committee of Usak University (ID:38824465-020), prospectively registered on www.clinicaltrials.gov (*NCT04713813*). The study was administered based on the updated Consolidated Standards of Reporting Trials (CONSORT) statement and the CONSORT guidelines were followed.

Prior to a participant’s recruitment, all subjects completed informed consent forms. Also, as a part of the consent process all volunteers were told that they could quit the study at any time as part of the consent process.

**Figure 1.**
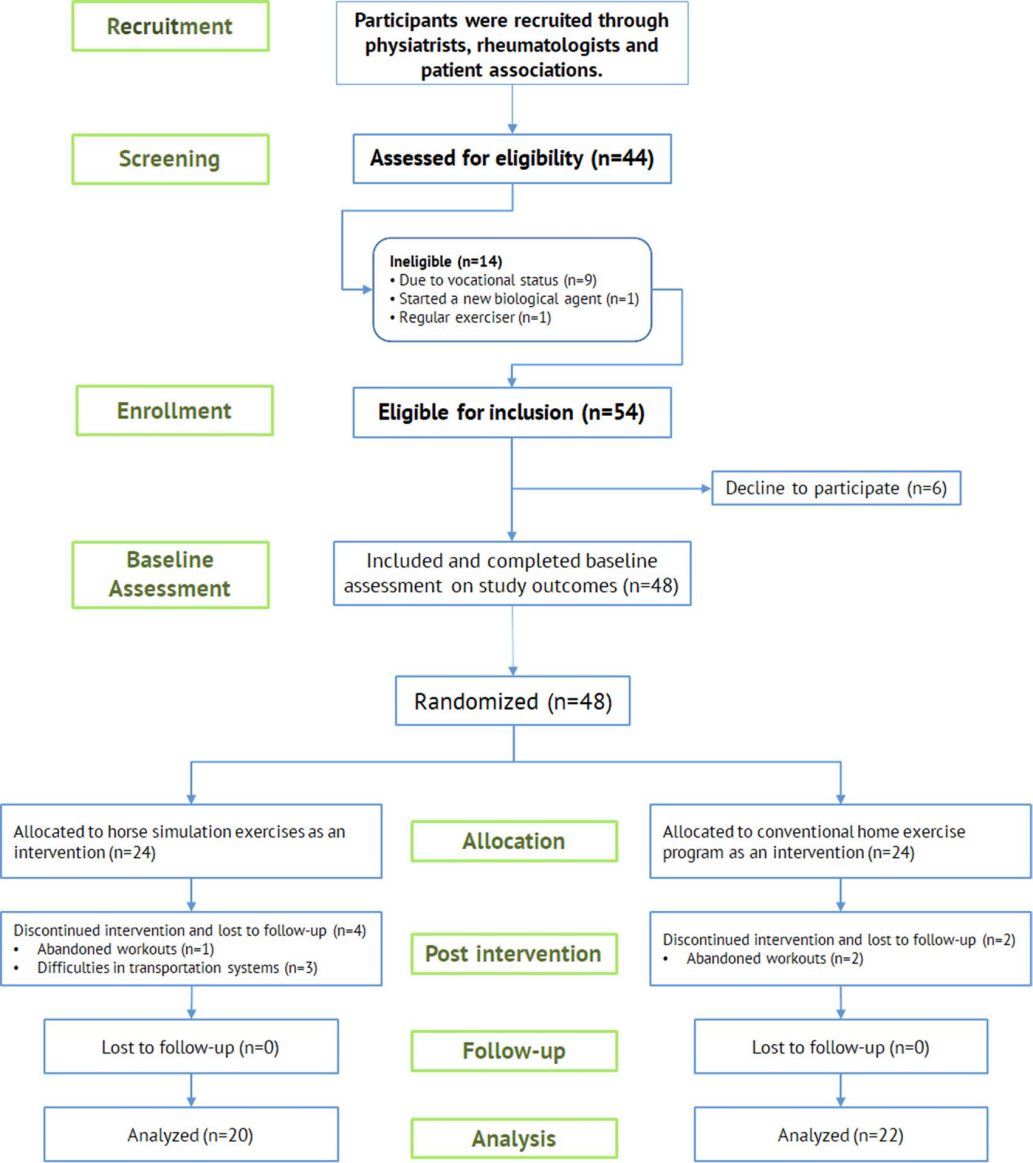
Study flow diagram.

Participants were recruited through the announcements on social media and newsletter from the local AS patient associations and by word of mouth among the outpatient clinic staff of the rheumatology department. Individuals were recruited and enrolled based on the following inclusion criteria: confirmed diagnosis of AS by a rheumatologist according to the modified New York diagnostic criteria, diagnosed as AS at least for two years, age 18 to 50 years, regular use of disease-modifying anti-rheumatic drugs (anti-tumor necrosis factor (TNF) agents, sulfasalazine) at a stable dosage for at least twelve weeks. Patients were excluded if they had a regular exercise habit (totaling a minimum of 150 min of moderate exercise each week) during the previous six months, or in case of the presence of comorbid conditions including severe heart, kidney and respiratory disease, also, any problems that may prevent exercising (mental health, orthopedic, and neurological). The dropout criteria for this study were serious dosage changes in regular medication or a new treatment strategy during the study period, and continuity to exercise sessions of less than 85% (see Figure 1).

### Randomization and blinding

Participants who met the inclusion criteria were randomized to HS or conventional home (CH) exercise groups. A statistician blinded to the study prepared the computerized random allocation sequence with different block sizes. Furthermore, to ensure that gender and age were similarly represented, random assignment to the intervention arms was stratified according to these variables.

There were three measurement periods: the baseline, after the interventions (12th week), and the 24th week long-term follow-up (following the removal of interventions). So, the same physiotherapist (an outcome assessor) who assesses the patient status and outcome measures was blinded to study protocols. Baseline and follow-up assessments lasted between 45 and 60 min.

### Interventions

Participants in both exercise groups received 48 exercise sessions (40 min each), that is 4 sessions a week for twelve consecutive weeks. In both types of exercise, each session consisted of a five-minute warm-up, including side lunges, side stepping, half squat, biceps, triceps and deltoid warm-up exercises, and self-range of motion exercises for the cervical, thoracic and lumbar spine. In addition, 5 min of static stretches (including ‘downward facing dog’, ‘seated straddle’, ‘cat-cow’, ‘standing calf stretch’ and ‘upper trapezius stretch’ positions) were performed in both groups at the end of the exercise.

**Figure 2.**
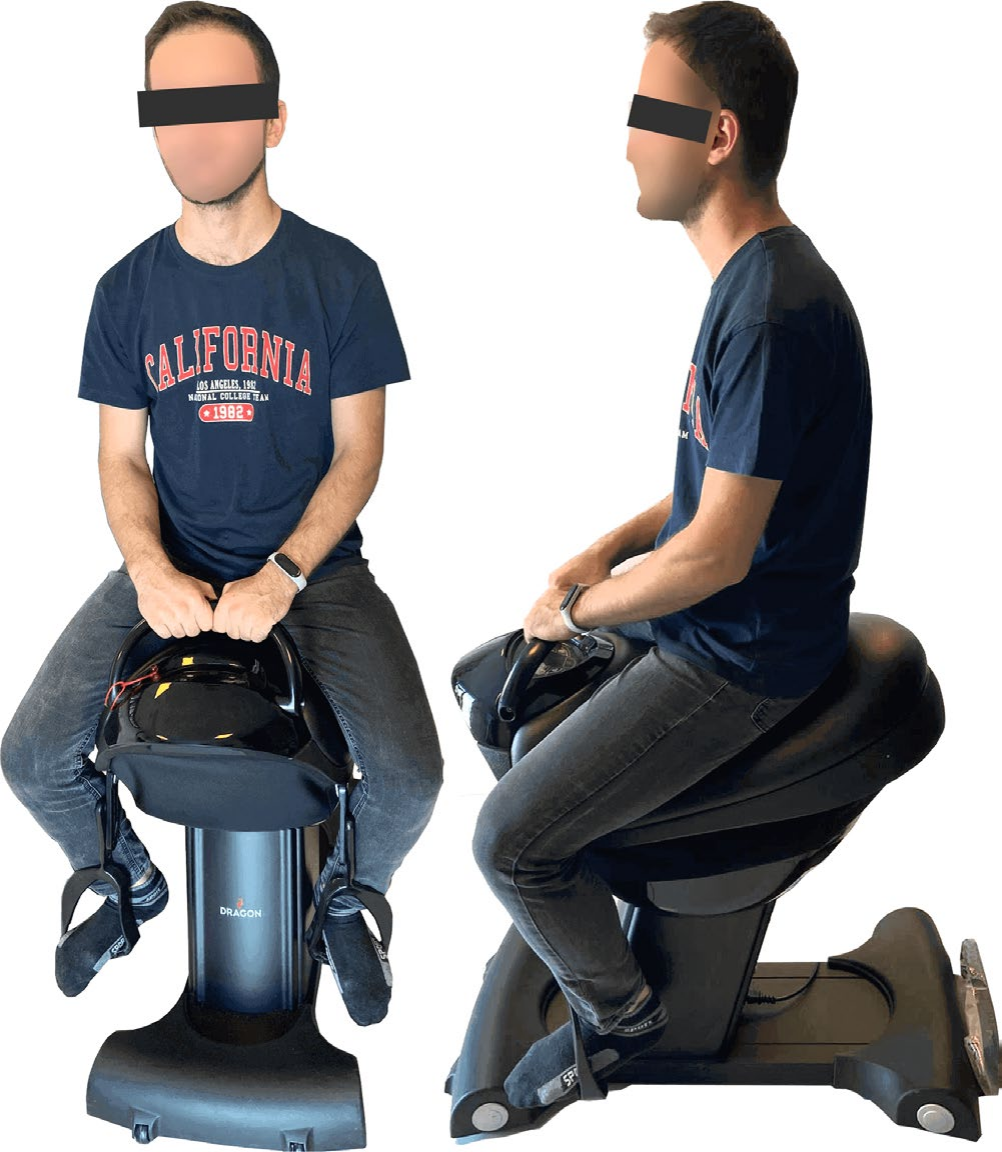
(a and b) A Patient’s position for the horse stimulation exercise using the Dragon® hippotherapy simulator.

**Figure 3.**
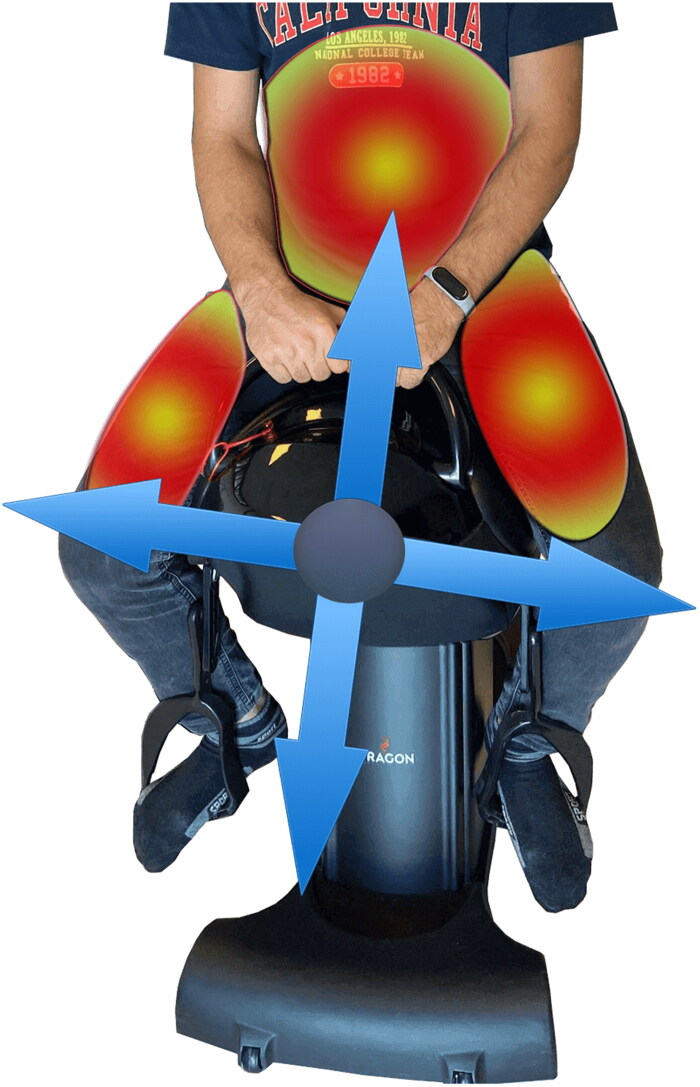
Muscle groups that are active to ensure correct posture and balance are shown while the simulator is running.

Patients in the HS exercise group used the Dragon Hippotherapy Simulator (Code TA-022) as gym equipment and performed their exercise sessions in the gymnasium of XXX University Hospital. An experienced physiatrist (AYK) supervised the sessions to ensure that the patients performed the exercises correctly. The Dragon was built for indoor use and has a three-phase motor to mimic the three-dimensional movements of a live horse (up and down, forward and backward, and left to right). Participants sit in a leather-covered saddle and hold on to the saddle’s horn-like handles. Sitting in the simulator, the participant’s feet in the stirrups are in dorsiflexion, both knees and hips are in semiflexion and the hip joint is in semi abduction and external rotation. While the device was in motion, participants were verbally reminded to keep their head upright and look forward and to maintain correct posture (see Figures 2 and 3). They were also motivated to keep their shoulders up to provide the necessary position to maintain balance. The sessions continued by increasing the speed of the device according to the patient’s ability.

**Figure 4.**
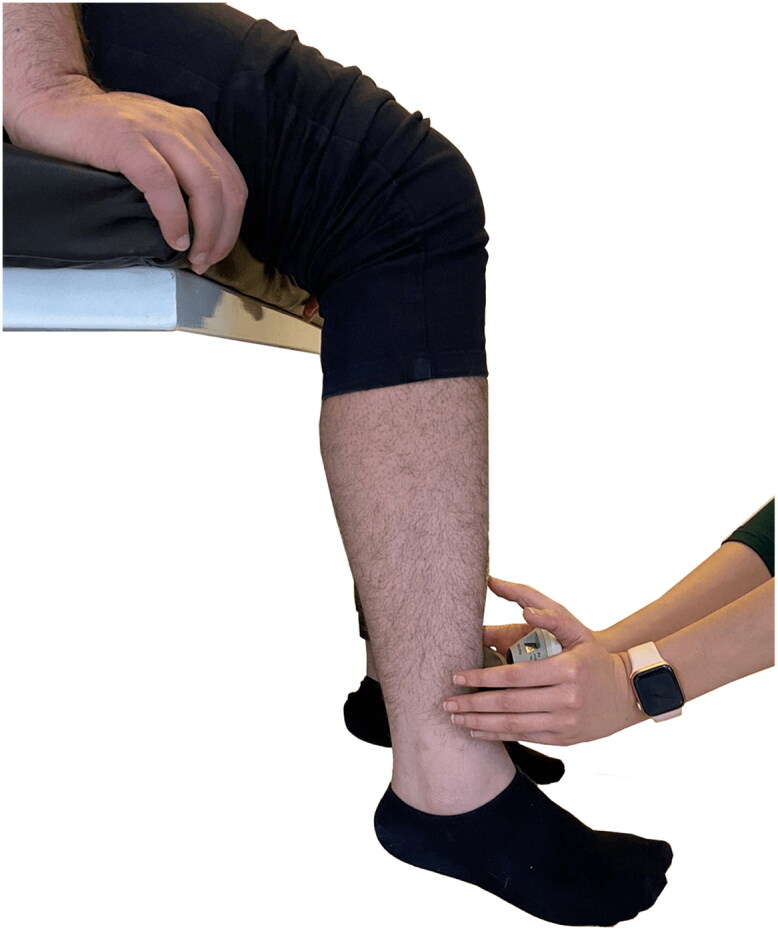
Position of patients with ankylosing spondylitis for the microFET® 2 dynamometer knee-extensor assessment.

The conventional home exercise program consisted of a warm-up (mentioned above), body weight strength training, balance exercises, and static stretching (mentioned above), as demonstrated by the physiatrist (AYK) at the beginning of the study period. During the study period, participants were encouraged to participate and their continuity was checked by phone calls within 14 days. In addition, all patients received an exercise manual booklet and exercise diary.

> Low- or moderate-intensity bodyweight exercise routines were physical tasks performed in the current study. The first exercise consisted of 6–8 knee or elevated push-ups, followed by 8–12 assisted bodyweight squats, 8–12 walking or jumping jacks, and finally 8–12 assisted lunges. The participants then performed 8–12 repetitions of leg drops, scissor kicks, and chin tucks (with towel support) in the prone position. The rest was allowed between movements, but the participants were required to complete each set of each exercise before starting a new round.

>Simple, enjoyable and safe balance exercises were selected for people with AS to learn. Sessions began with a standing march and 3-way kicks for 20–30 s, followed by a one-leg stand (supported if necessary) for up to 30 s. Finally, heel-to-toe standing or walking was performed for up to 30 s. A set of 3 repetitions was performed.

### Outcome measurements

For a comprehensive analysis, outcome measures were chosen to document changes in muscle strength, disease activity, spinal mobility and quality of life across different components of the International Classification of Functioning, Disability and Health, including activity, body structure and function. Primary outcomes included quadriceps muscle strength, Bath Ankylosing Spondylitis Disease Activity Index (BASDAI), Bath Ankylosing Spondylitis Functional Index (BASFI), Bath Ankylosing Spondylitis Metrology Index (BASMI) and Ankylosing Spondylitis Quality of Life Scale (ASQoL).

Patients were evaluated by an experienced physiotherapist (the evaluator was blinded to study design and group allocation) at baseline, post-intervention and after 6 months of follow-up. Measurements were taken at the same time of day (10:00 am) and measured in order (muscle test, BASDAI, BASFI, BASMI and ASQoL) in the same room. The physiotherapist allowed sufficient rest time between tests to avoid patient fatigue.

### Quadriceps muscle strength testing

Quadriceps muscle strength was tested with a microFET-2 dynamometer. Reliability and quantifiable force evaluation tests (Hogan Health Industries, Inc., UT, USA) were performed with this portable (wireless) handheld dynamometer [26]. With clinician stabilization, this device has previously demonstrated good-to-excellent inter- and intra-rater reliability and concurrent validity for a knee strength test in both seated and prone positions.

The subject was seated in an upright position with arms folded across the chest and no lumbar support. A small gap was left between the back of the knees and the chair and the knees were positioned at the same height as the hips (see Figure 4.). The patient was asked and encouraged to perform maximal isometric contractions of the quadriceps muscle for 5 s while the physiotherapist produced a counter horizontal resistance force. Three isometric maximal voluntary contractions were maintained from the dominant side (with 90 s’ recovery time between each assessment) and the most successful force attempt was recorded in Newtons (N).

### BASDAI

The patient was asked to answer six questions regarding the five main symptoms of AS (fatigue, spinal pain, joint pain, tenderness, pain intensity and morning stiffness) over the past week. These self-administered questions are on a 0–10 scale and the resulting score of 0 to 50 is divided by five to obtain the final BASDAI score (0–10). Low BASDAI scores indicate low disease activity and good disease control, while high scores indicate high disease activity and poor disease control [27].

### BASFI

Ten questions comprising the BASFI were asked to assess patients’ activities related to their functional and anatomical limitations. A ten-point visual analog scale (zero being ‘easy’ and ten being ‘impossible’) was used to score each question, and the BASFI score (a value between 0 and 10) was obtained from the average of the ten scales. Higher values are indicative of severe impairment [28].

### BASMI

The BASMI was used to assess the effect of exercise interventions on spinal mobility. This scale ranges from 0 to 10, with lower scores indicating less mobility limitation and 10 indicating very severe limitation. Four measures of spinal mobility (tragus-to-wall distance, cervical rotation, lumbar lateral flexion and modified Schober’s test) and one measure of hip mobility (intermalleolar distance) were applied. The total score was calculated using the BASMI-10 scoring system [29].

### ASQoL

The 18-item Ankylosing Spondylitis Quality of Life (ASQoL) instrument was used as a reliable tool to assess the impact of exercise interventions on pain, mood, sleep, motivation and ability to cope with daily tasks, relationships, self-image and self-esteem. Higher scores on these Yes/No questions indicate poorer quality of life in AS patients [30].

### Sample size calculation

The number of participants included in this study was determined based on quadriceps muscle test data from a previously published study. According to the results of Ogut et al. the mean muscle strength before treatment was 40.6 Nm with a standard deviation of 6.5. The mean muscle strength after treatment was 44.8 Nm. The sample size calculation was based on a power of 80% (β 0.2), a dropout rate of 25% and a statistical significance (α = 0.05) of 95% (*p* = .05). Therefore, 24 patients were required in each group.

### Statistical analysis

Data analysis was performed using IBM® SPSS® statistics 17.0 software (SPSS Inc. Released 2008. SPSS Statistics for Windows, Version 17.0. Chicago). The normal distribution of variables was examined using the Kolmogorov-Smirnov test, which was suitable for our sample sizes. Median, minimum and maximum values and percentages were used to present quantitative data. Changes from baseline to follow-up examinations are presented descriptively as trend directions in the outcome response. Change within group was assessed by calculating the change in variables (follow-up value minus baseline value) and reported as median difference. Student’s t-test was used to compare baseline demographic and clinical characteristics between groups and descriptive statistics were calculated for demographic data. Cohen’s d coefficient was used to calculate the effect size for change in each group (mean SD of differences). Effect sizes were divided into three categories: small ‘0.20–0.49’, medium ‘0.50–0.80’ and high ‘>0.8’. A P-value less than 0.05 was considered statistically significant.

## Results

Forty-eight people with AS participated in this study. Six patients dropped out due to inability to participate in exercise sessions (*n* = 4) and inadequate follow-up (*n* = 2). Finally, 42 of the 48 participants completed the post-intervention assessment (month 3) and follow-up at 6 months (Figure 1).

The mean age for the entire cohort was 34.9 years and 64.2% of the cohort was male. The groups were similar and there was no significant difference between the two groups in terms of demographic/clinical characteristics and baseline physical examination findings (Table 1) (*p* > .05). Compliance with exercise sessions was calculated as the percentage of workouts attended by each participant. Compliance with the conventional home-based exercise program was 85.4% for the CH group (mean 41.21 of 48 possible sessions; min 40; max 48) and 87.5% for the HS group (mean 42.32 of 48 possible sessions; min 41; max 48). There was no significant difference between the two groups in terms of compliance rates (*p* = .251).

**Table 1.** Demographics and characteristics of participants (*n* = 42).

Characteristics	HS group (*n* = 20)	CH group (*n* = 22)	*p*
Age (y) mean ± SD	34.4 ± 7.3	35.5 ± 7.3	.612
Sex n (%)			.2.45
Female	7 (35%)	8 (36.3%)	
Male	13 (65%)	14 (63.6%)	
BMI mean ± SD	28.4 ± 4.5	27.6 ± 5.1	.116
Education (y) mean ± SD	13.2 ± 5.5	13.1 ± 4.6	.354
Duration of symptoms (months)	72.4 ± 36.4	76.4 ± 42.1	.211
Age at initial diagnosis	28.4 ± 5.8	28.6 ± 6.3	.114
Medications *n* (%)			.342
NSAIDs only	6 (30%)	6 (27.2%)	
Sulfasalazine and NSAIDs	6 (30%)	7 (31.8%)	
Biologic medications	8 (40%)	9 (40.9%)	
Smoking Rate	11 (55%)	12 (54.5%)	.123

HS: Hippotherapy simulation exercise; CH: Conventional home exercise; SD: Standard deviation; BMI: body mass index (kg/m^2^); y: year; *n*: number; NSAID: non-steroidal anti-inflammatory drugs.

No serious or mild adverse effects were noted in either group. Only one patient experienced transient discomfort immediately after the first two sessions of HS exercise. However, this patient tolerated the exercise well and no medical intervention was required.

Baseline BASDAI, BASFI, BASMI, ASQoL and muscle strength scores were similar between the two groups (*p* > .05) (Table 2). Under exercise conditions and from baseline to week 12, BASDAI, BASFI, BASMI, ASQoL and muscle strength scores improved significantly in both groups (*p* < .05). BASDAI, BASFI and BASMI scores decreased significantly in the HS group compared to the CH group at week 12 (*p* = .005, *p* = .003 and *p* = .045, respectively), but there was no significant difference between the two groups in terms of ASQoL and muscle strength scores at week 12 (*p* = .245 and *p* = .212, respectively). However, as seen in Table 2, intergroup comparison did not reveal a significant difference between the two groups for all outcome measures at 3-month follow-up (*p* > 0.05). The results of between- and within-group analyses and effect sizes are presented in Table 2.

**Table 2.** Within-group and between group comparisons of outcomes.

				Pre- to Post Intervention		Pre intervention to Follow-up		‘p’ value for difference	
	Pre intervention (Mean ± SD)	Post intervention (Mean ± SD)	Follow-up (Mean ± SD)	‘p’ value	Effect size	‘p’ value	Effect size	Post int	Follow-up
Muscle strength (N)								.212	.177
HS	250.8 ± 64.5	271.2 ± 67.6	251.3 ± 62.1	.001	0.30	.264	0.07		
CH	255.4 ± 71.4	279.2 ± 75.4	253.6 ± 63.9	.001	0.31	.457	0.02		
BASDAI								.005	.072
HS	5.2 ± 1.6	3.1 ± 1.3	4.7 ± 1.4	.006	0.95	.341	0.27		
CH	5.2 ± 1.6	3.9 ± 1.4	5.1 ± 1.7	.009	0.91	.555	0.14		
BASFI								.003	.079
HS	3.6 ± 1.4	1.6 ± 1.1	3.4 ± 1.4	.019	0.78	.311	0.35		
CH	3.7 ± 1.5	2.9 ± 1.5	3.7 ± 1.5	.020	0.83	.375	0.28		
BASMI								.045	.321
HS	2.8 ± 1.4	1.7 ± 1.1	2.3 ± 1.4	.015	0.42	.247	0.08		
CH	2.8 ± 1.5	2.0 ± 1.2	2.3 ± 1.5	.039	0.46	.313	0.08		
ASQoL								.245	.267
HS	10.6 ± 3.6	6.6 ± 2.7	9.9 ± 3.4	.022	0.55	.135	0.60		
CH	10.9 ± 3.7	7.2 ± 3.0	10.6 ± 3.6	.025	0.50	.156	0.63		

BASDAI: Bath Ankylosing Spondylitis Disease Activity Index; BASFI: Bath Ankylosing Spondylitis Functional Index; BASMI: Bath Ankylosing Spondylitis Metrology Index; ASQoL: Ankylosing Spondylitis Quality of Life Scale N: newton; HS: Hippotherapy simulation exercise; CH: Conventional home exercise; SD: Standard deviation.

## Discussion

This study demonstrated that 48 sessions of hippotherapy simulation workouts significantly improved functionality, muscle strength and quality of life in individuals with AS leading a sedentary lifestyle. Comparing the treatment approaches, daily activity scores, functionality and spinal mobility showed a statistically better improvement in the HS group at week 12. All patients tolerated the planned exercise programs and no adverse events were recorded during the training sessions in either group. Nevertheless, this prospective study did not show improvement in any study outcome at 6-month follow-up.

According to the analytical assessment of Hilliere et al. HS improves the physical and mental skills of elderly patients [31]. They also emphasize that hippotherapy and simulation sessions both significantly improve physical function in older people from pre to post intervention [31]. We anticipate that future work will address HS exercises in the elderly population, which could be a safe and sustainable exercise plan in the group of AS patients with bone changes/ankylosis, mobility and balance disorders, osteoporosis, and cardiorespiratory consequences of the disease. The movements of the horse are used in occupational therapy techniques to improve motor control, coordination, balance, attention, sensory processing and performance in daily activities. Therefore, we hypothesized that HS exercises contribute to spinal mobility, disease activity, and functionality through similar mechanisms. Sustainability of physical activity promotes and supports overall health, well-being and functional outcomes. According to our results, adherence rates for the exercise session were high in both groups (85.4% for the CH group and 87.5% for the HS group). The riding simulator has significant advantages over live horse therapy, which has an older and evidenced use in rehabilitation. The simulator is lightweight, low-priced, has no space restrictions and is not affected by weather conditions. It also promotes balance and postural stability exercises in a safe and non-excessive way [23–25]. Therefore, these technological features increase participation in the exercise program offered by the simulator.

Potential reasons for heterogeneity in exercise trials on people with AS include participants’ differing disease severity, exercise frequency, intensity, duration and types of exercise. However, the results of systematic reviews of exercise interventions in patients with AS show a high level of consistency, suggesting mild to moderate benefits for disease activity, axial mobility, function and pain. According to a Cochrane review aimed at evaluating the benefits of exercise programs for people with AS, exercise approaches showed a reduction in disease activity with low-quality evidence compared with usual care [6]. Furthermore, for physical function, measured by the BASFI scale, moderate-quality evidence showed an improvement after exercise sessions, and a clinically meaningful improvement was observed for spinal mobility with very-low-quality evidence [6]. However, according to the results of the Cochrane review, no clinically meaningful benefit was seen for the BASFI and BASMI scales immediately after completion of the exercise program [6]. However, in this study, more favorable improvements were seen in both groups immediately after completion of the exercise interventions. Thus, based on the findings of our study under exercise conditions and from baseline to week 12, BASDAI, BASFI and BASMI scores were significantly reduced in the HS group compared to the CH group (*p*=.011, *p*=.013 and *p*=.045, respectively). Three different clinical trials evaluating the effectiveness of exercise programs in patients with ankylosing spondylitis (versus no intervention) found significantly lower disease activity, improved physical function score, and increased spinal mobility at the end of the exercise intervention [32–34]. Similarly in our study, both exercise groups showed significant improvements in BASDAI, BASFI and BASMI scores at the end of the interventions (week 12), but no improvement was seen at long-term follow-up (week 24). Few studies have evaluated quality of life as an outcome of exercise interventions in the AS population. Bestas et al. and Dundar et al. emphasized that water-based exercise showed more remarkable effects on improvement in quality of life than land-based exercise [35,36]. Garcia et al. found the effects of exercises on quality of life to be insufficient [37]. According to our results, improvement in disease scores was achieved with HS and exercises may improve quality of life in AS patients. Beinotti et al. found significant improvement in SF-36 total score after HS exercises in a stroke population and suggested that HS exercises may provide positive quality of life outcomes for people with stroke [38]. Similar results regarding quality-of-life outcomes were reported by Silkwood-Sherer DJ and McGibbon NH in children with cerebral palsy [39]. Therefore, we anticipate that further clinical research will be conducted to demonstrate that HS exercises may be an effective intervention to treat musculoskeletal deficits, movement abnormalities and quality-of-life outcomes in diverse patient populations.

A few AS studies have included muscle strength training as part of the exercise program. However, in most of these studies, neither muscle strength nor other relevant physiological responses were measured [40]. In a study by Carter et al. evaluating the physical abilities of patients with AS, researchers found that participants’ weak quadriceps and other peripheral muscles were mostly responsible for their reduced aerobic capacity and exercise intolerance [41]. Two studies by Sahin et al. evaluating ankle and knee muscle strength and fatigue in AS patients along with healthy controls provided evidence of lower extremity weakness in AS patients [42,43]. Souza et al. evaluated the effectiveness of a progressive muscle strengthening program using a Swiss ball for AS patients and reported increased muscle strength in the abdominal, quadriceps and triceps muscles. Measurement of muscle strength with a hand-held dynamometer is a strong point of our study method and our results showed that HS exercises performed for 12 weeks increased quadriceps muscle strength in AS patients [33]. Kim and Lee examined muscle activation of core muscles in an elderly population and reported significant improvement in rectus abdominis, erector spinae, quadratus lumborum, external oblique and gluteus medius muscle strength with a 40-session HS exercise-based intervention [44]. Our findings are consistent with the study of Kim and Lee, who observed non-significant changes in muscle strength during long-term follow-up periods.

A home-based exercise approach is a simple and cost-effective treatment for AS patients. Compared to a control group, there is moderate-quality evidence that home-based or supervised individualized exercise regimens have a positive impact on various measures of spinal mobility in AS patients [40]. Aytekin et al. investigated the effects of home-based exercise therapy on disease activity, pain, quality of life and mobility in AS patients. With range of motion, stretching, strengthening, posture and breathing exercises, the exercise group showed significant improvements in pain, mobility, quality of life and disease activity scores at the third month [45]. In our study, we applied a similar home exercise program for the control group and reported comparable improvement in muscle strength, BASDAI, BASMI and BASFI scores.

Although this study provided novel and important results, some potential shortcomings should be mentioned. While acute-phase reactants, particularly erythrocyte sedimentation rate (ESR) and C-reactive protein (CRP), may be quantitative follow-up parameters that can be used to monitor AS patients, our study did not have laboratory tests to define disease activity or progression with interventions. Second, in comparing the two separate exercise regimens, the groups were given the same amount of time to exercise but not the same intensity. Therefore, future studies should compare AS patients who received HS treatment with AS patients who received supervised exercise on land or in water. Another limitation is that we can only generalize our results to individuals with AS with mild disabilities; therefore, caution should be exercised in generalizing the results. Finally, not all measures were collected because of concerns about survey overload. Future studies should include assessment of balance and core muscle activation or endurance. Cost-effectiveness and treatment satisfaction with HP are also issues that need to be investigated.

## Conclusion

The results of this first randomized clinical trial of HS exercises for AS patients indicate a positive effect on disease activity, quality of life and muscle strength. Thus, the social advantage of this clinical trial is the opportunity to introduce a novel recreational exercise approach for AS patients. The possibility of combining HS exercises with other types of therapy could be a new research topic in future studies.

## References

[1] Braun J, Sieper J. Ankylosing spondylitis. Lancet. 2007;369(9570):1–11. doi: 10.1016/S0140-6736(07)60635-7. 17448825

[2] Mauro D, Thomas R, Guggino G, et al. Ankylosing spondylitis: an autoimmune or autoinflammatory disease? Nat Rev Rheumatol. 2021;17(7):387–404. doi: 10.1038/s41584-021-00625-y. 34113018

[3] Ambarus C, Yeremenko N, Tak PP, et al. Pathogenesis of spondyloarthritis: autoimmune or autoinflammatory? Curr Opin Rheumatol. 2012;24(4):351–358. doi: 10.1097/BOR.0b013e3283534df4. 22488076

[4] Millner JR, Barron JS, Beinke KM, et al. Exercise for ankylosing spondylitis: an evidence-based consensus statement. Semin Arthritis Rheum. 2016;45(4):411–427. doi: 10.1016/j.semarthrit.2015.08.003. 26493464

[5] Zochling J, van der Heijde D, Burgos-Vargas R, et al. ASAS/EULAR recommendations for the management of ankylosing spondylitis. Ann Rheum Dis. 2006;65(4):442–452. doi: 10.1136/ard.2005.041137. 16126791 PMC1798102

[6] Regnaux JP, Davergne T, Palazzo C, et al. Exercise programmes for ankylosing spondylitis. Cochrane Database Syst Rev. 2019;10: CD011321. 31578051 10.1002/14651858.CD011321.pub2 PMC6774752

[7] Karahan AY, Tok F, Yildirim P, et al. The effectiveness of exergames in patients with ankylosing spondylitis: a randomized controlled trial. Adv Clin Exp Med. 2016;25(5):931–936. doi: 10.17219/acem/32590. 28028958

[8] Kocyigit BF, Sagtaganov Z, Yessirkepov M. The effectiveness of yoga as a form of exercise in the management of rheumatic diseases. Rheumatol Int. 2023;43(5):795–801. doi: 10.1007/s00296-023-05291-9. 36856817

[9] Boudjani R, Challal S, Semerano L, et al. Impact of different types of exercise programs on ankylosing spondylitis: a systematic review and meta-analysis. Disabil Rehabil. 2022;11:1–12. doi: 10.1080/09638288.2022.2140842. 36369692

[10] Ortolan A, Webers C, Sepriano A, et al. Efficacy and safety of non-pharmacological and non-biological interventions: a systematic literature review informing the 2022 update of the ASAS/EULAR recommendations for the management of axial spondyloarthritis. Ann Rheum Dis. 2023;82(1):142–152. doi: 10.1136/ard-2022-223297. 36261247

[11] Medrado LN, Mendonca MLM, Budib MB, et al. Effectiveness of aquatic exercise in the treatment of inflammatory arthritis: systematic review. Rheumatol Int. 2022;42(10):1681–1691. doi: 10.1007/s00296-022-05145-w. 35633390

[12] Hosseini M, Rahimibarghani S, Ghorbanpour S, et al. The effects of supervision on the outcomes of exercise training in patients with ankylosing spondylitis: a single-blind randomized controlled trial. Int J Rheum Dis. 2023;26(6):1120–1128. doi: 10.1111/1756-185X.14711. 37096931

[13] Altan L, Korkmaz N, Dizdar M, et al. Effect of pilates training on people with ankylosing spondylitis. Rheumatol Int. 2012;32(7):2093–2099. doi: 10.1007/s00296-011-1932-9. 21499876

[14] Cetin SY, Calik BB, Ayan A, et al. The effectiveness of 10-Tai chi movements in patients with ankylosing spondylitis receiving anti-tumor necrosis factor α therapy: a randomized controlled trial. European J Integr Med. 2020;39:101208. doi: 10.1016/j.eujim.2020.101208.

[15] Sarac DC, Bayram S, Tore NG, et al. Association of core muscle endurance times With balance, fatigue, physical activity level, and kyphosis angle in patients With ankylosing spondylitis. J Clin Rheumatol. 2022;28(1):e135–e40. doi: 10.1097/RHU.0000000000001641. 33252392

[16] Sterba JA, Rogers BT, France AP, et al. Horseback riding in children with cerebral palsy: effect on gross motor function. Dev Med Child Neurol. 2002;44(5):301–308. doi: 10.1111/j.1469-8749.2002.tb00815.x. 12033715

[17] Park ES, Rha DW, Shin JS, et al. Effects of hippotherapy on gross motor function and functional performance of children with cerebral palsy. Yonsei Med J. 2014;55(6):1736–1742. doi: 10.3349/ymj.2014.55.6.1736. 25323914 PMC4205717

[18] Koca TT, Ataseven H. What is hippotherapy? The indications and effectiveness of hippotherapy. North Clin Istanb. 2015;2(3):247–252. 28058377 10.14744/nci.2016.71601 PMC5175116

[19] Kim SG, Lee CW. The effects of hippotherapy on elderly persons’ static balance and gait. J Phys Ther Sci. 2014;26(1):25–27. doi: 10.1589/jpts.26.25. 24567669 PMC3927035

[20] Lindroth JL, Sullivan JL, Silkwood-Sherer D. Does hippotherapy effect use of sensory information for balance in people with multiple sclerosis? Physiother Theory Pract. 2015;31(8):575–581. doi: 10.3109/09593985.2015.1067266. 26467902

[21] Meregillano G. Hippotherapy. Phys Med Rehabil Clin N Am. 2004;15(4):843–854, vii. doi: 10.1016/j.pmr.2004.02.002. 15458756

[22] Lee CW, Kim SG, Na SS. The effects of hippotherapy and a horse riding simulator on the balance of children with cerebral palsy. J Phys Ther Sci. 2014;26(3):423–425. doi: 10.1589/jpts.26.423. 24707098 PMC3976017

[23] Kubota M, Nagasaki M, Tokudome M, et al. Mechanical horseback riding improves insulin sensitivity in elder diabetic patients. Diabetes Res Clin Pract. 2006;71(2):124–130. doi: 10.1016/j.diabres.2005.06.012. 16105705

[24] Yu CH, Hong CU, Kang SR, et al. Analysis of basal physical fitness and lumbar muscle function according to indoor horse riding exercise. Biomed Mater Eng. 2014;24(6):2395–2405. 25226940 10.3233/BME-141053

[25] Salbaş E, Karahan AY. Effects of hippotherapy simulation exercise vs. conventional home exercises on muscle strength and balance in people with multiple sclerosis: a randomized controlled trial. Mult Scler Relat Disord. 2022;68:104111. doi: 10.1016/j.msard.2022.104111. 36031694

[26] Larson D, Lorenz D, Melton B. Can clinician-stabilization with hand-held dynamometry yield a reliable measure of knee flexion torque? Int J Sports Phys Ther. 2022;17(6):1095–1103. 36237659 10.26603/001c.37907 PMC9528694

[27] Garrett S, Jenkinson T, Kennedy LG, et al. A new approach to defining disease status in ankylosing spondylitis: the bath ankylosing spondylitis disease activity ındex. J Rheumatol. 1994;21(12):2286–2291. 7699630

[28] Calin A, Garrett S, Whitelock H, et al. A new approach to defining functional ability in ankylosing spondylitis: the development of the bath ankylosing spondylitis functional ındex. J Rheumatol. 1994;21(12):2281–2285. 7699629

[29] Jenkinson TR, Mallorie PA, Whitelock HC, et al. Defining spinal mobility in ankylosing spondylitis (AS). The bath AS metrology ındex. J Rheumatol. 1994;21(9):1694–1698. 7799351

[30] Duruoz MT, Doward L, Turan Y, et al. Translation and validation of the turkish version of the ankylosing spondylitis quality of life (ASQOL) questionnaire. Rheumatol Int. 2013;33(11):2717–2722. doi: 10.1007/s00296-013-2796-y. 23765201

[31] Hilliere C, Collado-Mateo D, Villafaina S, et al. Benefits of hippotherapy and horse riding simulation exercise on healthy older adults: a systematic review. Pm R. 2018;10(10):1062–1072. doi: 10.1016/j.pmrj.2018.03.019. 29626616

[32] Sveaas SH, Berg IJ, Provan SA, et al. Efficacy of high intensity exercise on disease activity and cardiovascular risk in active axial spondyloarthritis: a randomized controlled pilot study. PLoS One. 2014;9(9):e108688. doi: 10.1371/journal.pone.0108688. 25268365 PMC4182541

[33] Souza MC, Jennings F, Morimoto H, et al. Swiss ball exercises improve muscle strength and walking performance in ankylosing spondylitis: a randomized controlled trial. Rev Bras Reumatol Engl Ed. 2017;57(1):45–55. doi: 10.1016/j.rbr.2016.04.008. 28137402

[34] Masiero S, Bonaldo L, Pigatto M, et al. Rehabilitation treatment in patients with ankylosing spondylitis stabilized with tumor necrosis factor inhibitor therapy: a randomized controlled trial. J Rheumatol. 2011;38(7):1335–1342. doi: 10.3899/jrheum.100987. 21459942

[35] Bestaş E, Dündar Ü, Köken T, et al. The comparison of effects of balneotherapy, water-based and land-based exercises on disease activity, symptoms, sleep quality, quality of life and serum sclerostin level in patients with ankylosing spondylitis: a prospective, randomized study. Arch Rheumatol. 2022;37(2):159–168. doi: 10.46497/ArchRheumatol.2022.9024. 36017205 PMC9377174

[36] Dundar U, Solak O, Toktas H, et al. Effect of aquatic exercise on ankylosing spondylitis: a randomized controlled trial. Rheumatol Int. 2014;34(11):1505–1511. doi: 10.1007/s00296-014-2980-8. 24626605

[37] Fernandez Garcia R, Sanchez Sanchez LC, Lopez Rodriguez MM[, et al. Effects of an exercise and relaxation aquatic program in patients with spondyloarthritis: a randomized trial]. Med Clin (Barc). 2015;145(9):380–384. doi: 10.1016/j.medcle.2016.03.001. 25639496

[38] Beinotti F, Correia N, Christofoletti G, et al. Use of hippotherapy in gait training for hemiparetic post-stroke. Arq Neuropsiquiatr. 2010;68(6):908–913. doi: 10.1590/s0004-282x2010000600015. 21243251

[39] Silkwood-Sherer DJ, McGibbon NH. Can hippotherapy make a difference in the quality of life of children with cerebral palsy? A pragmatic study. Physiother Theory Pract. 2022;38(3):390–400. doi: 10.1080/09593985.2020.1759167. 32406798

[40] Dagfinrud H, Halvorsen S, Vollestad NK, et al. Exercise programs in trials for patients with ankylosing spondylitis: do they really have the potential for effectiveness? Arthritis Care Res (Hoboken). 2011;63(4):597–603. doi: 10.1002/acr.20415. 21452270

[41] Carter R, Riantawan P, Banham SW, et al. An investigation of factors limiting aerobic capacity in patients with ankylosing spondylitis. Respir Med. 1999;93(10):700–708. doi: 10.1016/s0954-6111(99)90036-7. 10581658

[42] Sahin N, Ozcan E, Baskent A, et al. Muscular kinetics and fatigue evaluation of knee using by isokinetic dynamometer in patients with ankylosing spondylitis. Acta Reumatol Port. 2011;36(3):252–259. 22113600

[43] Sahin N, Ozcan E, Baskent A, et al. Isokinetic evaluation of ankle muscle strength and fatigue in patients with ankylosing spondylitis. Eur J Phys Rehabil Med. 2011;47(3):399–405. 21364512

[44] Kim SG, Lee JH. The effects of horse riding simulation exercise on muscle activation and limits of stability in the elderly. Arch Gerontol Geriatr. 2015;60(1):62–65. doi: 10.1016/j.archger.2014.10.018. 25465508

[45] Aytekin E, Caglar NS, Ozgonenel L, et al. Home-based exercise therapy in patients with ankylosing spondylitis: effects on pain, mobility, disease activity, quality of life, and respiratory functions. Clin Rheumatol. 2012;31(1):91–97. doi: 10.1007/s10067-011-1791-5. 21656347

